# Biotransformation of selected secondary metabolites by *Alternaria* species and the pharmaceutical, food and agricultural application of biotransformation products

**DOI:** 10.1007/s13659-024-00469-5

**Published:** 2024-08-19

**Authors:** Babalwa Tembeni, Olusola Emmanuel Idowu, Rachid Benrkia, Salima Boutahiri, Opeyemi Joshua Olatunji

**Affiliations:** African Genome Center, Mohammed VI Polytechnic University, Benguerir, Morocco

**Keywords:** *Alternaria*, Biotransformation, Fermentation, Cytochrome P450, Cofactor-NADPH

## Abstract

Biotransformation is a process in which molecules are modified in the presence of a biocatalyst or enzymes, as well as the metabolic alterations that occur in organisms from exposure to the molecules. Microbial biotransformation is an important process in natural product drug discovery as novel compounds are biosynthesised. Additionally, biotransformation products offer compounds with improved efficacy, solubility, reduced cytotoxic and allows for the understanding of structure activity relationships. One of the driving forces for these impeccable findings are associated with the presence of cytochrome P450 monooxygenases that is present in all organisms such as mammals, bacteria, and fungi. Numerous fungal strains have been used and reported for their ability to biotransform different compounds. This review focused on studies using *Alternaria* species as biocatalysts in the biotransformation of natural product compounds. *Alternaria* species facilitates reactions that favour stereoselectivity, regioselectivity under mild conditions. Additionally, microbial biotransformation products, their application in food, pharmaceutical and agricultural sector is discussed in this review.

**Graphical Abstract:**

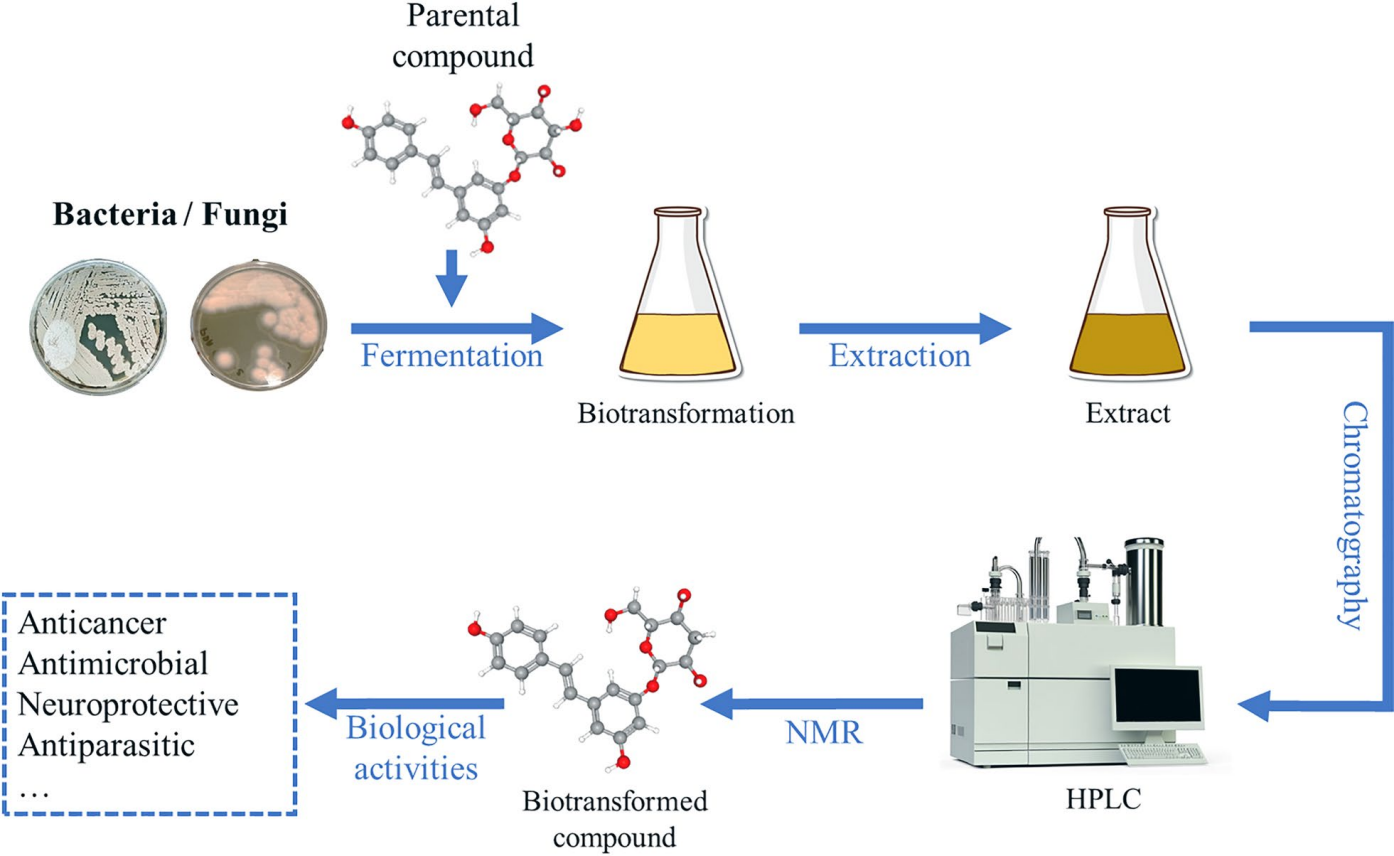

## Introduction

Medicinal plants are reservoirs of structurally diverse compounds with a plethora of biological activities ranging from anti-microbial, cytotoxicity, anti-viral, anti-inflammatory activity, anti-diabetic and more [1–4]. However, their isolation reflects challenges faced in the lab as they are often isolated in minute quantities [1, 5–8]. It is also noteworthy to always remember the significant contributions these compounds have given to humankind as several drugs in the market owe their structural backbone to a natural product compound [9–12]. Also, any modification on a natural product or its total synthesis takes away its natural state, as the conditions required to synthesise them are harsh due to complex stereochemistry and stereocenters they possess [13–15]. In nature, there are different pathways that take place to obtain a single pure compound, however each class of compound has its specific pathway requiring precursors such as acetyl-CoA, geranylgeranyl diphosphate (GGPP), isopentenyl diphosphate (IPP) and its isomer dimethylallyl diphosphate (DMAPP) for their biosynthesis [16].

Upon discovery and isolation, the physico-chemical properties of these drug-like molecules exhibited bioavailability issues, solubility, and toxicity, hence their development in drug discovery has been halted. These amongst other challenges faced in natural product drug discovery, alternatives such as semisynthesis of natural product to modify the chemistry and introduce or eliminate some moieties have been made [13, 17–19]. However, synthetic routes to obtain stereoselective or regioselective chemical entities under mild conditions is challenging [20–22]. These challenges opened doors for the use of microbial organisms leading to biotransformation products [23, 24].

Microbial biotransformation reactions are chemical reactions catalysed by microorganisms such as fungi, bacterial or enzymes and occur in two phases [21, 23]. The reactions inactivate and improve the hydrophobic nature of compounds to produce more hydrophilic derivatives [21]. The process detoxifies compounds by either hydroxylation, hydrogenation or dehydrogenation on the parent compound to promote excretion of less harmful products [14]. Moreover, biotransformation products have been reported to possess greater pharmacological activity than the parent compound [25]. In some cases, the activity of biotransformation products may be unrelated to the parent compound, while in other cases the activity may be enhanced or destroyed all together [8, 26, 27].

Biotransformation studies have been proven to be an effective and environmentally friendly alternative in the synthesis of natural products requiring high regioselectivity and stereoselective reaction settings as most reactions are carried out under mild reaction conditions such as ambient temperature [26, 28, 29]. The regio and stereoselective nature of microbial reactions has resulted in several enantiomers with good activity while maintaining the “natural” state of a compound, which cannot be said with semi-synthesized derivatives [29, 30]. Moreover, microbial biotransformation is an efficient tool to be exploited in predicting xenobiotic drug metabolism as the enzymes are like those present in mammals [17, 19]. Numerous microbial strains have been reported and these include *Alternaria* fungal strains.

*Alternaria* species are pathogenic fungi found in fruits as black spots, some *Alternaria* species are reported to possess negative effects in human health as they cause asthma and other life-threatening diseases due to the mycotoxins, they produce [31–34]. *Alternaria* mycotoxins are generally classified into three groups namely dibenzopyrone derivatives, perylene derivatives and tetramic acid derivatives [35]. The dibenzopyrone derivatives include compounds such as alternariol, alternariol monomethyl ether, and altenuene [36], while the perylene derivative include altertoxins-I, altertoxins-II and altertoxins II. The tetramic acid derivative include compounds such as tenuazonic acid [35, 36]. However, other classes of compounds have been isolated from various *Alternaria* species and have been discussed in detail in numerous studies [31, 36–38].

Antibacterial agents have been used to fight against these fungal strains, but due to their ability to biotransform compounds as a survival tactic, some interesting compounds exhibiting wonderful pharmacological application were obtained [39–41]. In an experiment where an *Alternaria* species was treated with anti-fungal agents belonging to norsesquiterpenes and terpenes, the fungi were able to reduce the anti-fungal effects of the compounds by modifying them into less toxic compounds as the compounds underwent hydroxylation, carboxylation, and dihydroxylation reactions [38, 42]. Additionally, the ability of *Alternaria* species to undergo this remarkable altering/biotransformation properties is mainly due to the presence of cytochrome P450 monooxygenases (P450s) system which are known to facilitate oxidation reactions in microbial biotransformation reactions [43–46].

Several reviews report the use of microbial organisms in the biotransformation of natural product compounds, for example, the biotransformation of clerodanes, pimaranes, abietanes, trachylobanes, kaurenes, beyeranes, and stemodanes has been carried out using numerous microorganisms with no mention of *Alternaria* species [47]. Other reviews focused on the chemo-enzymatic transformation of taxanes and their reversal activity towards MDR tumor cells and only mentioned the genus *Alternaria* [43, 48, 49]. The same trend is observed in the review focusing on the biotransformation of diterpenoids by microorganisms [50]. Hence this review focuses on the biotransformation of natural products with *Alternaria* species as a biotransformation catalyst and the application of biotransformation compounds in food, agriculture, and pharmaceuticals.

## Microbial mediated stereoselective biotransformation reactions

Microorganisms function as biocatalysts in the development of novel drug leads by producing compounds with functionalized moieties due to their ability to facilitate controlled oxidation reactions in C − H bonds of compounds [13, 21, 43, 47, 48]. One of the sought-after biocatalysts used include those possessing P450s which *Alternaria* species possess [26–28, 45]. These biocatalysts have the potential of oxidizing sp^3^ and sp^2^ C − H bonds with a high degree of chemo, regio, and stereoselectivity and other compounds with varying complexities in their structures [29, 30]. The enzymes are also responsible for the metabolic breakdown of drugs in humans, the biodegradation of pesticides, and environmental pollutants [28, 29]. The monooxygenation on sp^3^ and sp^2^ hybridized carbons is usually denoted by oxidation, epoxidation, methylation reactions amongst others, while the C = C double bonds oxidation is one of the widely known oxidation reactions by P450s [29, 45].

P450s are a large group of hemeprosthetic monooxygenase enzymes linked to a protein [29]. The enzymes are responsible for a plethora of oxidative reactions against highly diverse range of substrates. They activate molecular oxygen (O_2_), by simultaneously incorporating one oxygen atom into an organic substrate, while producing a water molecule with the remaining oxygen atom as shown in Eq. 1[51]. This mechanism uses the universal cellular cofactors NADH or NADPH as a reducing agent to deliver electrons via iron-sulfur protein or via flavoprotein [49, 51].

Reaction 1 $${\text{NAD}}\left( {\text{P}} \right){\text{H}}\,\,{ + }\,\,{\text{O}}_{2} \to {\text{NAD}}\left( {\text{P}} \right)\, + \,{\text{RO}}\,{ + }\,{\text{H}}_{2} {\text{O}}$$

Reaction 2 $${\text{O}}_{{2}} + {\text{R}} \to {\text{RO}}_{2}$$

Reaction 3 $${\text{O}}_{2} {\mkern 1mu} + {\mkern 1mu} {\text{R}}{\mkern 1mu} + {\mkern 1mu} {{R^{\prime}}} \to {\text{RO}}{\mkern 1mu} + {\mkern 1mu} {{R^{\prime}O}}$$

**Equation **1**:** Stoichiometric reaction in P450s facilitated oxidation reaction [51].

**Fig. 1:**
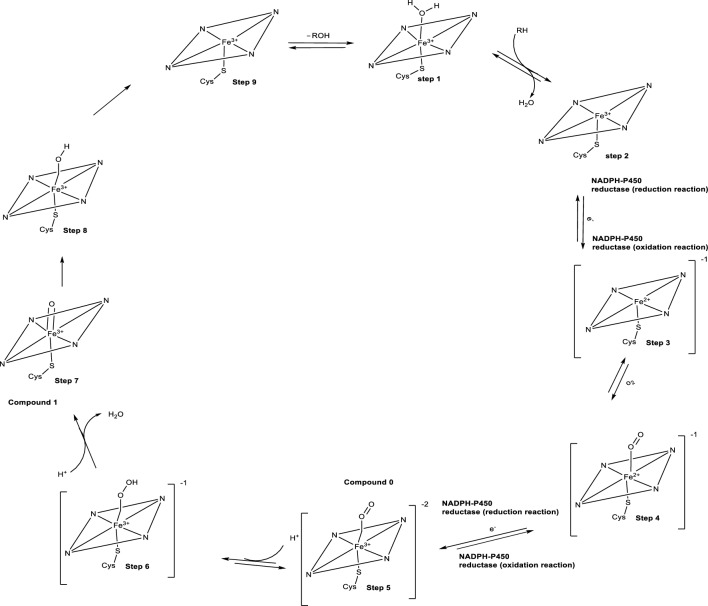
Catalytic Cycle of Cytochrome P450s. [27, 45, 51, 52]

Reports on the mechanisms involved in the oxidation of molecules using P450s to understand the reactivity and regioselectivity properties of these enzymes are widely available [45, 51]. Also, many studies have been conducted to understand the mechanisms involved in the catalytic cycle of P450s [45]. Briefly (Fig. 1), step 1 shows how the substrate is indirectly bound near the inactive heme iron. Step 2 shows the delivery of electrons by a di-flavin reductase followed by the binding of molecular oxygen to the inactive ferrous iron to form a Fe–O complex (step 3). Step 4 shows the formation of a Fe_3_^+^-O_2_¯ complex also known as compound 0. The formation of complex 0 is associated with the delivery of electrons from the reductase and is widely postulated to be involved in certain oxidation reactions. Step 5 and 6 involves the protonation of compound 0, and H_2_O elimination from the complex to obtain compound 1 previously described as FeO^3+^. The unstable compound 1 is mainly involved in most oxidation reactions involving P450s. Step 7 shows how compound 1 when near a substrate, is responsible for a hydrogen atom removal/ elimination from the substrate. Oxygen rebound occurs in step 8 via radical recombination to afford an oxygenated molecule and the formation of an enzyme–substrate complex taking place in step 9 [27, 45, 51, 52].

**Fig. 2:**
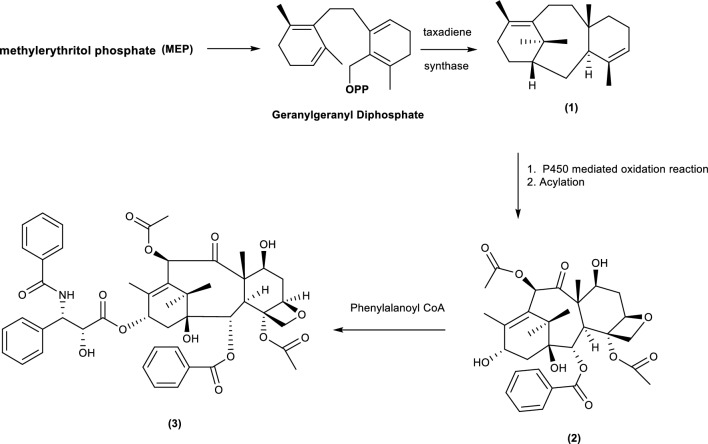
Biotransformation of a taxane derivative via a P450s mediated reaction to afford paclitaxel. [53, 54]

The efficiency of these enzymes, compared with chemical methods, in catalysing the insertion of oxygen into unactivated C − H bonds under mild reaction conditions has sparked interest among researchers toward investigating and exploiting P450s for a variety of synthetic applications [26, 29, 30]. The capacity to which these enzymes can perform to biosynthesise molecules is highly dependent on the substrates used [53, 54]. Below is a proposed route to which P450s produce biotransformation products. The illustration below makes use of the known taxane compound paclitaxel from beccatin under the same conditions as described in Fig. 2.

### Oxygenation reactions

**Fig. 3:**
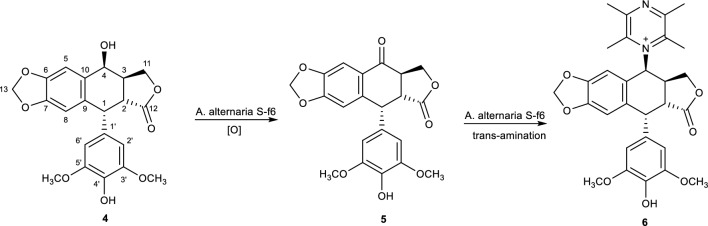
Oxygenation reaction facilitated by *A. alternata* [55]

Most oxygenation reactions during microbial transformation take place in phase I. Where a substrate-inducible oxygenase in the presence of molecular oxygen and the cofactor NADPH allows the insertion of a reactive oxygen atom at specific positions on the substrate to produce oxygenated molecules. Additionally, this reaction produces unstable intermediates that undergo spontaneous nonenzymatic rearrangement such as the elimination of acetyl groups, methyl migration to produce compounds with different functionalities as end products. An example includes the microbial biotransformation of 4′-demethylepipodophyllotoxin **(4)** in the presence of *A. alternata* S-f6 as a transformation catalyst to biosynthesise 4′-demethylpodophyllotoxone (DMEP) **(5)**. The fungi facilitated the conversion of the hydroxyl group at position C-4 to a carbonyl group. Additionally, the activating agent C = O present in DMEP subsequently underwent trans-amination with ligustrazine after incubation with an *Alternaria* species resulting in the formation of 4-(2,3,5,6-tetramethylpyrazine-1)-4′-demethylepipodophyllotoxin (4-TMP-DMEP) **(6)** as shown in Fig. 3.[55]

As stated by many authors that biotransformation products have the capacity to exhibit elevated bioactivity compared to the parent compound, so in comparing the bioactivity of biotransformation products with the parent compound, the biotransformed products were reported with a 50% effective concentration (EC_50_) that was more than 5000 folds efficacious than **(4)** (EC_50 =_ 529 μM) and **(5)**- (EC_50_ = 0.11 μM). Simultaneously, the EC_50_ of **(6)** against the normal human proximal tubular epithelial cell line HK-2 (i.e., 0.40 μM) was 66 times higher than that of **(4)** (i.e., 0.006 μM). Furthermore, compared with the parent compound **4** (i.e., log *P* = 0.34), the water solubility of biotransformation product **(6)** (i.e., log *P* = 0.66) was significantly enhanced by 94% [55].

**Fig. 4:**
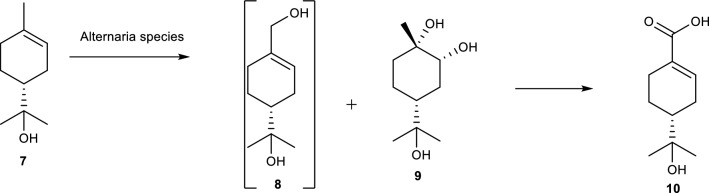
The biotransformation of α-terpenol by *A. alternata* to produce two oxidative products namely 4*R*-oleuropeic acid (**9)** and (1*S*,2*R*,4*R*)-*p*-menthane-1,2,8-triol (**10**). [56]

The anti-fungal and anti-bacterial monoterpene α-terpinol **7**, a volatile component of numerous pharmaceutical preparations, is another compound of interest in biotransformation studies. The metabolic pathway of **(7)** in mammals has been investigated with reports showing P450s as reaction catalysts. Additionally, biotransformation capabilities of *A. alternata* with **7** produced two oxidative products namely 4*R*-oleuropeic acid **(9)** and (1*S*,2*R*,4*R*)-*p*-menthane-1,2,8-triol **(10)** as shown in Fig. 4. [56] The biotransformation resulting to **(9)** is reported as a one step process while the reaction for **(10)** is via an intermediate 7-Hydroxy-α-terpineol **(8)** [56].

There is no available data on the bioactivity of **(9)** yet, but its derivatives are reported to exhibit anti-bacterial, anti-inflammatory, and analgesic properties. Additionally, the biotransformation of **7** is shown to be stereoselective with observed stereochemistry on **(9)** and an unstable diol intermediate that is later acetylised to form **(10)** [57].

**Fig. 5:**
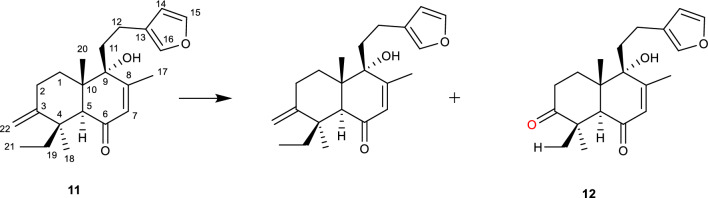
Biotransformation of solidagenone using *A. alternata* to yield 3-oxosolidagenone **(12)** [40]

Solidagenone **(11)** isolated for the first time from the rhizomes of *Solidago chilensis* Meyen (Asteraceae) is reported to have potent proliferative properties against different cancer cell lines [57]. Additionally, the gastroprotective properties and low oral toxicity of **(11)** led to further studies with focus on the structure activity relationship of the compound along with its derivatives [40, 58]. The biotransformation of **(11)** in the presence of *A. alternata* ATCC 44501 led to the production 3-oxosolidagenone **(12)** [40, 58]. The biotransformation was reported to be regioselective as the oxygenation took place at position C-3 of the molecule as shown in Fig. 5. The reaction is notably initiated by ring A hydroxylation with no report on the formed intermediate [40].

The bioactivity of **12** is attributed to the furan moiety [58]. Additionally, derivatives possessing a hydroxyl group at position C-3 also possess some form of bioactivity. Some more examples of compounds that are reported to have experienced oxygenation mediated reaction in the presence of an *Alternaria* species are listed in Table 1.

**Table 1:** Examples of compounds undergoing oxygenation reactions. For each substrate the specific *Alternaria* species used, and site of reaction is presented

Substrate	*Alternaria* species	Biotransformed products	Refs.
Oleandrin	*A. eureka*	7(β)-hydroxyoleandrin, 7(β)-hydroxy,16-desacetyloleandrin, 7(β)-hydroxy,16- desacetylnerigoside,	[59]
Cycloastragenol [20(*R*),24(*S*)-epoxy-3β,6α,16β,25-tetrahydroxycycloartane	*A. eureka*,	16-oxo-20(27)-octanor-cycloastragenol, 11(β)-hydroxy,3,16-dioxo-20(27)-octanor-cycloastragenol, (2α*R*,5α*S*,5β*S*,7*S*,7α*R*,9*S*,11α*R*,12α*S*)-7,9-dihydroxy-2α,5α,8,8-tetramethyltetradecahydro-4H,12H-cyclopenta[α]cyclopropa [e]phenanthren-4-one, (3*S*,5*R*,6*S*,8*S*,11*S*,13*R*,14*S*,16*R*)-11-(hydroxymethyl)-4,4,13,14-tetramethyl-2,3,4,5,6,7,8,11,12,13,14,15,16,17- tetradecahydro-1H-cyclopenta[α]phenanthrene-3,6,16-triol and (1*S*,2α*R*,5α*S*,5β*S*,7*S*,7α*R*,11α*R*,12α*R*)-1,7-dihydroxy-2α,5α,8,8-tetramethyldodecahydro-4H,12H-cyclopenta[α]cyclopropa [e]phenanthrene-4,9(5H)-dione,	[60]
Rutalexin	*A. brassicicola*	Phomapyrone G, rutapyrone,	[61]
oleanolic acid	*A. longipes*	2α,3α,19α-trihydroxy-ursolic acid-28-*O*-β-d-glucopyranoside, 2α,3β,19α-trihydroxy-ursolic acid-28-*O*-β-*d*-glucopyranoside, oleanolic acid 28-*O*-β-d-glucopyranosyl ester, oleanolic acid-3-*O*-β-d-glucopyranoside, 3-*O*-(β-d-glucopyranosyl)-oleanolic acid-28-*O*-β-d-glucopyranoside, 2α,3β,19α-trihydroxy-oleanolic acid-28-*O*-β-d-glucopyranoside	[33]
oleanolic acid	*A. alternata AS 3.4578*	1β,3β,16α-trihydroxy-olean-11,13(18)-dien-28β-oic acid, 1β,3β,16α-trihydroxy-olean-12-en- 28β-oic acid, 3β,16α,29-trihydroxy-olean-12-en-28β-oic acid, 3-oxo-16α-hydroxy- olean-12-en-28β-oic acid	[62]

### Hydroxylation reactions

**Fig. 6:**
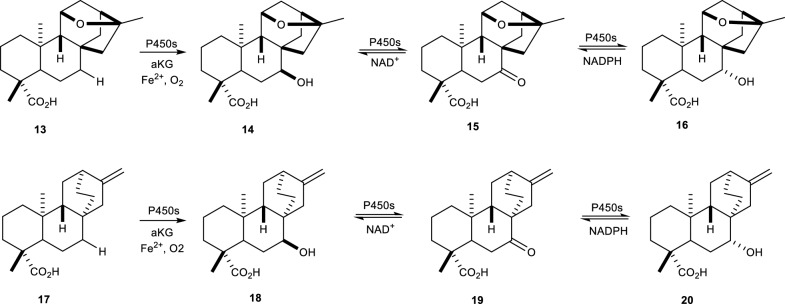
Stereospecific hydroxylation mechanism facilitated by P450s [63]

Enzymatic hydroxylation reactions involve the bioconversion of a C-H bond to form a C–OH bond, with the assistance of the hydrolase enzymes [30, 53, 63]. The isolation of hydroxylase enzymes is reported to be a challenging process due to their unstable nature in pure form [27–29, 64]. However, the use of microorganisms possessing P450s is known to facilitate the hydroxylation of natural products [28]. A study on the stereospecific hydroxylation of platensimycin and its biosynthesis reportedly shows how P450s facilitated reactions results to a C-7 α-OH orientation via a dehydrogenase installation as shown in Fig. 6 [63].

**Fig. 7:**
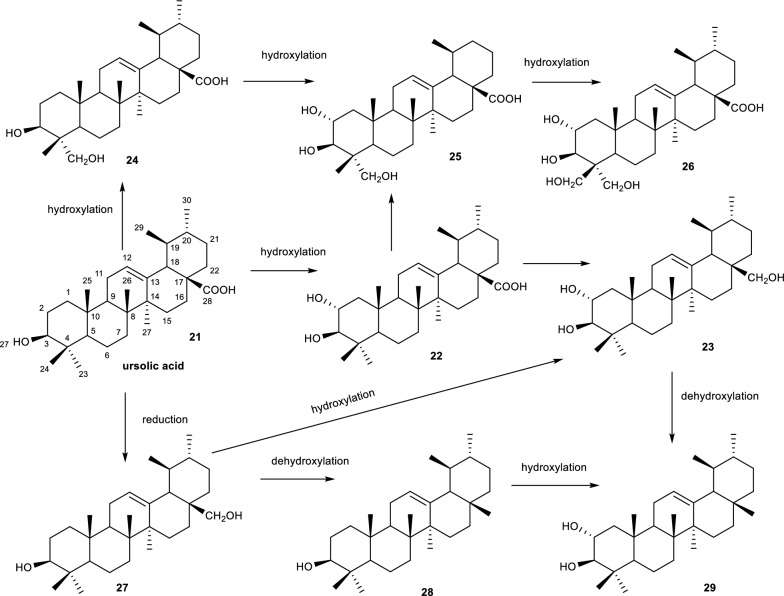
Proposed biotransformation of ursolic acid by *A. alternata*

Ursolic acid **(21)** a pentacyclic triterpenoid with medicinal properties including anti-inflammatory, analgesic and anti-cancer activities. The compound is also known for its water solubility problems, hence numerous selective biotransformation studies on **(21)** have been done to improve its solubility properties [41]. A biotransformation of ursolic acid by *A. alternata* resulted in 8 biotransformation products namely corosolic acid **(22)**, urs-12-en-2α,3β,28-triol **(33)**, 3β,23-dihydroxyurs-12-en-28-oic acid **(24)**, 2α,3β,23-trihydroxyurs-12-en-28-oic acid **(25)**, 2α,3β,23,24-tetrahydroxyurs-12-en-28-oic acid **(26)**, 3β,28-dihydroxy-12-ursene **(27)**, urs-12-en-3β-ol **(28)**, and urs-12-en-2α,3β-diol **(29)** with some possessing a common hydroxyl group at position C-2 as shown in Fig. 7 [41]. Upon examining the compounds for their antiproliferative properties, the authors observed an increase in the activity when comparing the anti-proliferative properties of **(21)** with compounds having multiple hydroxyl groups. The observed increase in efficacy was later attributed OH groups at positions C-2,23 and 24.

**Fig. 8:**
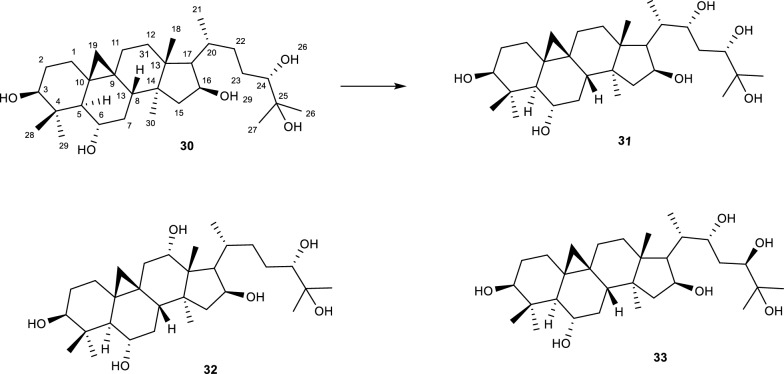
Biotransformation of cyclocanthogenol by *A. eureka* [66]

Cyclocanthogenol **(30)** is a cycloartane type triterpenoid, belonging to a class of compounds that possess anti-inflammatory, analgesic, sedative, and hypotensive properties [65]. The biotransformation of **(30)** by an *Alternaria* species afforded 8 compounds. However, only 3β,6α,12α,16β,24(*S*),25-hexahydroxycycloartane **(31)**, 3β,6α,16β,22,24(*S*),25-hexahydroxycycloartane **(32)** and 3β,6α,16β,17α,24(*S*),25-hexahydroxycycloartane **(33)** underwent hydroxylation [66]. The hydroxylation on the compounds was observed at different carbon positions C-12,17, and 22 as shown in Fig. 8. Notably, the stereochemistry of the compounds was not altered, implying that the enzyme did not facilitate the C-H bond rotation. Additionally, there is no record on the biological activity of the biotransformed compounds reported in this study [66].

**Fig. 9:**
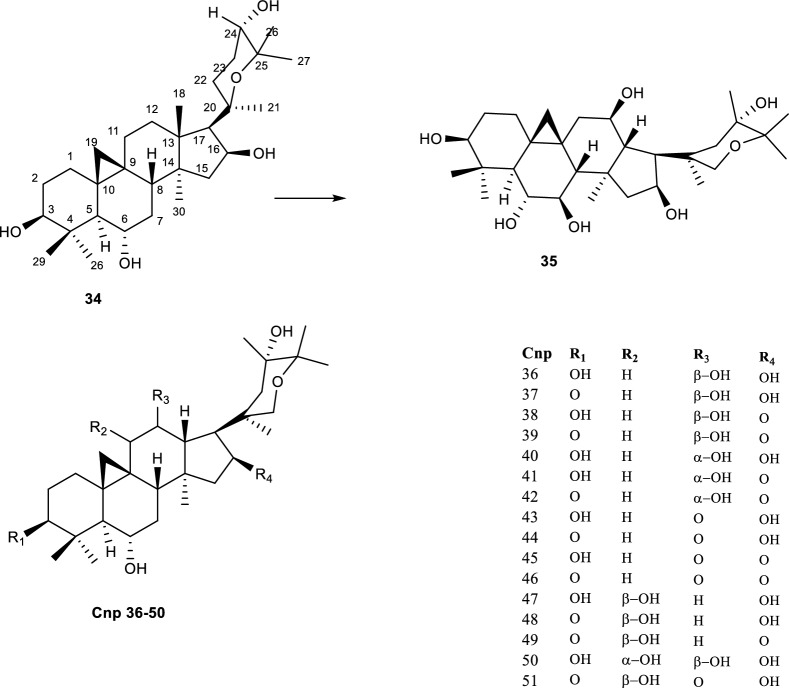
Biotransformation of cyclocephagenol with A. eureka 1E1BL1 [24]

Enhanced neuroprotective properties of a cycloartane-type sapogenin** (34)** were reportedly observed after incubating **(34)** with *A. eureka* 1E1BL1. The reaction afforded 17 biotransformation products **(35–51)** possessing hydroxyl groups in different carbon positions as shown in Fig. 9. Additionally, the hydroxylation on **(34)** mainly occurred at positions C-11 and/or C-12 for most compounds. A noticeable stereoselectivity of the reaction was observed, based on the α and β oriented hydroxyl groups reported on the molecules [24, 67]. Of the 17 biotransformation products only compound **(50)** possessed a hydroxyl group at both positions, also the orientation of the hydroxyl bonds was reported as α and β. The compounds were further reported to regulate the reduction of H_2_O_2_-mediated oxidative stress and inhibition of H_2_O_2_-induced mitochondrial damage [24]. Of particular interest about the biotransformation products of this reaction was the observed different reactions that were facilitated also included epoxidation, methyl migration, ring expansion, ring cleavage and so on [24, 67].

**Fig. 10:**
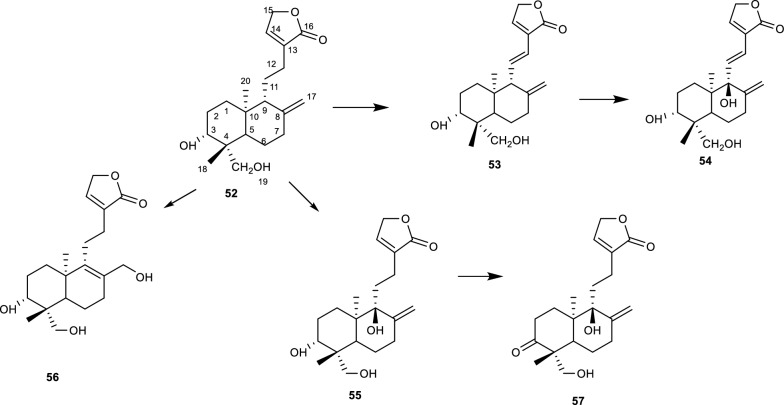
Biotransformation of deoxyandrographolide with *A. alternata* [32, 68]

The biotransformation of deoxyandrographolide **(52)** after incubation with *A. alternata* AS 3.4578 afforded 5 compounds namely dehydroandrographolide **(53)**, 9β-hydroxydehydroandrographolide **(54)**, 9β-hydroxy-deoxyandrographolide **(55)**, 3α, 17, 19-trihydroxyl-8, 13-ent-labdadien-15, 16-olide **(56)**, and 3-oxo-9β-hydroxy-deoxyandrographolide **(57)** as shown in Fig. 10. Of the 5 biotransformation products, **55–57** were reported as novel compounds, additionally the compounds possessed a β-oriented hydroxyl group at position C-9 [32, 68]. However, one of the molecules possessed an α-oriented hydroxyl group at position C-2. The addition reaction on the alkene group to afford the saturated hydroxyl side chain, is evidence that not only do *Alternaria* species catalyse reactions with sp^3^ hybridized carbon atoms but also those with sp^2^ hybridised carbon atoms. Studies on the cytotoxicity of the biotransformed products suggest that the C-9 hydroxylation improved the anti-cancer properties of the compounds [32, 68, 69].

**Fig. 11:**
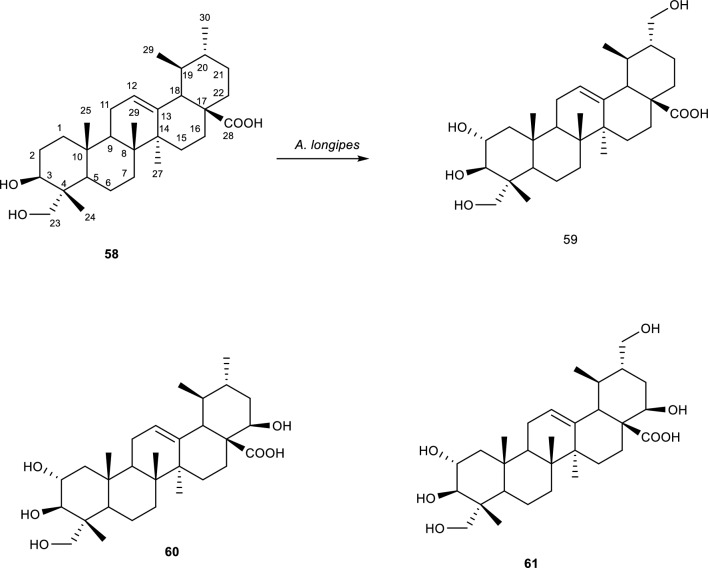
Biotransformation of *Asiatic acid* with *A. longipes* [70, 71]

Asiatic acid **(58)** is a major pentacyclic triterpene isolated from *Centella asiatica* and shows a variety of bioactive properties including anti-oxidative, anti-inflammatory, hepatoprotective, anti-Alzheimer’s, anti-depressant, and anti-cancer properties [70]. Due to these properties, interest in understanding the structure activity relationship of such compounds, multiple derivatives were obtained through biotransformation studies. After the incubation of **(58)** with *A. longipes,* three new derivatives namely 2α,3β,23,30-tetrahydroxyurs-12-ene-28-oic acid **(59)**, 2α,3β,22b,23-tetrahydroxyurs-12-ene-28-oic acid **(60)**, and 2α,3β,22β,23,30-pentahydroxyurs-12-ene-28-oic acid **(61)** were obtained as shown in Fig. 11. For compound **(59)** and **(61)** the hydroxylation reaction took place at C-30 and for compound **(60)** the hydroxylation took place at C-22. The hydroxylation at position C-22 afforded a molecule possessing an axial proton suggested that regioselective hydroxylation and stereoselectivity took place [70, 71].

Even though the compounds were biotransformed to investigate their structure activity relationship, the biological activity of these derivatives is not reported. However, other derivatives obtained through synthetic routes have been reported to exhibit good biological properties. Altering the compound at positions C-2, C-3, C-23, C-28 resulted in derivatives portraying good cytotoxic properties compared to the parent compound [70, 71]. Other biotransformation products formed using *Alternaria* species as a biocatalyst are listed in Table 2

**Table 2:** Examples of compounds undergoing hydroxylation reactions. For each substrate the specific *Alternaria* species used, and site of reaction is presented

Substrate	*Alternaria species*	Biotransformed products	Refs.
(-)-ambroxide	*A. alternata*	1β-hydroxyambroxide and 3β-hydroxyambroxide	[72]
Cycloastragenol	*A. eureka 1E1BL1*	16-oxo-20(27)-octanor-cycloastragenol, 11β-hydroxy,3,16-dioxo-20(27)-octanor-cycloastragenol and 1α-hydroxy-20(27)-octanor-cycloastragenol	[60]
Oleandrin	*A. eureka 1E1BL1*	7(β)-hydroxy oleandrin and β-hydroxy oleandrin	[73]
14-anhydrodigoxigenin	*A. alternata*	8β-hydroxy-14-anhydrodigoxigenone, 17α-hydroxy-14-anhydrodigoxigenone and 1β-hydroxy-14-anhydrodigoxigenone	[74]*)*
3-*epi*-desacetylcinobufagin	*A. alternata*	3-*epi*-12β-hydroxyl desacetylcinobufagin	[75]
Cinobufagin	*A. alternata*	12β- hydroxyl cinobufagin	[75]
Resibufogenin	*A. alternata*	12β-hydroxyl resibufogenin	[75]
Bufalin	*A. alternata*	12β-hydroxyl bufalin, 7β-hydroxyl Bufalin, 3-*oxo*-12β-hydroxyl bufalin and 3-*oxo*-7β-hydroxyl bufalin	[75]
Gitoxigenin	*A. eureka 1E1BL1*	7β-hydroxy-3-oxogitoxigenin, 3-dimethylacetal-7β-hydroxygitoxigenin, 3-oxodiginatigenin, 3-epidiginatigenin, 7β-hydroxy-3-oxo-gitoxigenin and 7β,12β-dihdyroxy-3-oxogitoxigenin	[39]
Grindelic acid	*A. alternata*	6-β-hydroxygrindelic	[54]

### Hydrogenation and dehydrogenation reactions

Generally, hydrogenation of natural products involves the reduction of a carbon–carbon double bond [76]. In biotransformation, a substrate with a carbon–carbon double bond or carbonyl group can undergo a hydrogenation reaction facilitated by microorganisms using the enzymes dehydrogenases, monooxygenases, dioxygenases, oxidases, peroxidases, or oxidoreductases [77]. Oxidoreductases catalyze the oxidation of primary and secondary amines, hydroxylation of aromatic or nonactivated carbon atoms, dehydrogenation of carbon–carbon single bonds, heteroatom oxygenation, Baeyer–Villiger oxidation, and double bond epoxidation [77].

Curcumin **(62)** is a major component of the rhizomes of *Curcuma longa* L. and is commonly used as a spice and for preserving food [78]. The compound belongs to the class of diarylheptanoid due to its 1,7-diphenylheptane carbon skeleton [79]. Curcumin has diverse biological activities such as antibacterial, antitumor, antioxidant, and hypoglycemic [78]. With all these benefits, curcumin's low bioavailability, poor water solubility, and structural instability make it difficult to use in pharmacological research. There are reports that the derivatives of curcumin are more potent than curcumin. Research involving the production of curcumin derivatives is on the increase lately, in which microbial biotransformation is inclusive [78, 79].

**Fig. 12:**
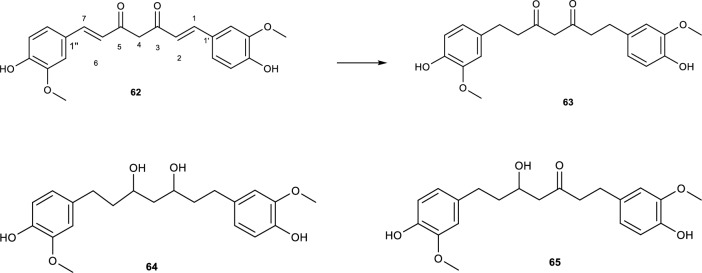
Biotransformation of curcumin with *A. alternata* AUMC 4685 [80]

Incubation of **(62)** with *A. alternata* AUMC 4685 produced three metabolites namely, tetrahydrocurcumin **(63)**, hexahydrocurcumin **(64)**, and octahydrocurcumin **(65)** [80]. The formation of metabolite (**63)** occurred by the hydrogenation of the diene moieties at position C-2 and C-6, while for metabolites **(64–65)**, the diene moieties were hydrogenated, and either one or both ketone moieties were reduced to a hydroxyl group as shown in Fig. 12 [78, 79]. The antioxidant, cytotoxic, and antimicrobial activities of these metabolites were also evaluated. The results of the antioxidant activity using the DPPH radical scavenging assay showed that metabolites **(63–65)** had radical scavenging activities with IC_50_ values better than curcumin.

The evaluation of the cytotoxic activity of these metabolites was done using an SRB assay and the results indicated a better cytotoxic activity of metabolites **(63–65)** against the Caco-2 cell line compared to curcumin. The antimicrobial activity of curcumin and metabolites **(63–65)** against *Staphylococcus aureus*, *Escherichia coli*, *Candida albicans*, and *Aspergillus niger* was tested using the agar well diffusion method. The results showed that these metabolites had higher antibacterial and antifungal activities against all the tested microorganisms than curcumin [80].

α-Phellandrene **(66)** is one of a pair of phellandrene cyclic monoterpene, and double-bond isomers. It is a constituent of the essential oil of *Boswellia sacra*, *Eucalyptus elata*, *Eucalyptus dives*, dill weed, and turmeric leaf. It is said that α-Phellandrene has a highly distinctive citrus, terpenic, slightly green, and black pepper-like smell, which is why it is frequently used in fragrances. α-Phellandrene has two enantiomers (–)-(R) and ( +)-(S), which have different olfactive and physicochemical properties [9, 10]. α-Phellandrene has been reported to possess several biological activities such as antioxidant, antitumor, anti-inflammatory, anti-nociception, and antimicrobial activities [81].

**Fig. 13:**
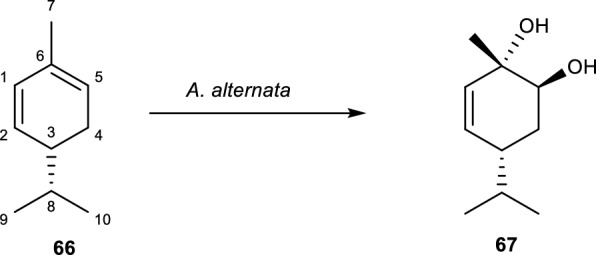
Biotransformation of α-Phellandrene with *A. alternata* [81, 82]

The biotransformation of **(66)** was performed using *A. alternata* to produce 5-*p*-menthene-1,2-diol **(66)** see Fig. 13 [82]. The formation of metabolite **(67**) occurred with the reduction of the C = C bond and the introduction of OH substituent at positions C-1 and C-2. The antimicrobial activity of metabolites **(**6**6)** and **(67)** was tested against some plant, human, and foodborne pathogens using the micro-dilution method [82]. The result indicated that **(66)** had a weak inhibition against all the tested bacteria at concentrations of 1 to > 4 mg/ml, while the antibacterial activity of metabolite **(67)** with MIC values of 0.125 to 0.5 mg/ml was better than that of metabolite **(66)**. The results of the antifungal activity of metabolites **(66)** and **(67)** against some *Candida* species showed that metabolite **(67)** had moderate inhibition. Metabolite **(67**) inhibited *Candida utilis* with good activity at a concentration of 0.125 mg/ml as compared to metabolite **(66)** [81, 82].

**Fig. 14:**
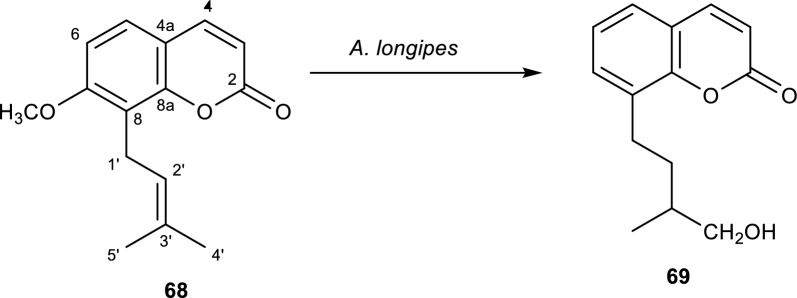
Biotransformation of osthole with *A. longipes* [37]

Osthole **(68)**, also referred to as osthol, is a derivative of coumarin [37]. It is present in some medicinal plants like *Cnidium monnieri* and *Angelica pubescens* [83]. The pharmacological activities of **(68)** have been shown to include antibacterial, hepatoprotective, immunomodulatory, neuroprotective, osteogenic, and cardiovascular protective properties [37, 83]. The incubation of 6**7** by *A. longipes* yielded one derivative. The derivative formed was identified as 4ʹ-hydroxyl-2ʹ,3ʹ-dihydroosthole **(69)** as shown in Fig. 14. The biotransformation occurred by the reduction of the carbon–carbon double bond at positions C-2ʹ and C-3ʹ [37].

**Fig. 15:**
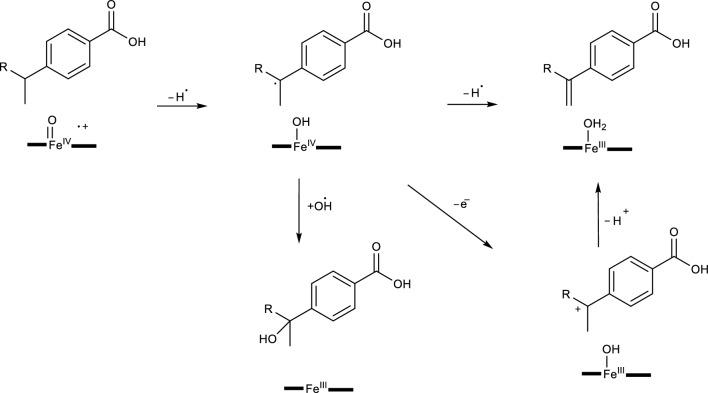
P450s mediated dehydrogenation reaction mechanism

P450s mediated dehydrogenation reactions are often overlooked compared to other common biotransformation reaction mechanisms [28, 45]. Due to this, there is very limited information on these reactions. Additionally, dehydrogenation reactions in biotransformation studies tend to produce toxic metabolites which can be factor to the limited studies observed [84]. However, some dehydrogenation products are known for their vital role in sterol biosynthesis. Currently, there are two possible mechanisms proposed for these reactions and they include a dehydrogenated product formed via two consecutive hydrogen abstractions. While the second proposed mechanism is said to be initiated after the occurrence of the first hydrogen abstraction to produce a radical intermediate, this is then followed by an electron transfer leading to the deprotonation of the resultant cation as shown in Fig. 15.

Cycloastragenol (**70**) is a cycloartan saponin of Astragaloside IV. It possesses a steroidal skeleton of tetracyclic triterpene. The compound was isolated from the dried roots of *Astragalus mongolica* and *Astragalus membranaceus*. **(70)** has a wide range of pharmacological properties, including liver protection, endothelial protection, pro-wound healing, anti-aging, anti-inflammatory, antibacterial, antiviral, and anti-fibrosis properties. Furthermore, **(70)** is the only telomerase activator known to exist in Traditional Chinese Medicine (TCM) extracts [60, 85].

**(70)** is thought to be a potential natural anti-aging agent. Based on the biological effects of the compound, its structural modification to find derivatives with increased anti-aging activity is significant. The structural diversity of triterpenoids is significantly influenced by microbial transformation [85] Feng et al. conducted the biotransformation of **(70)** using *A. alternata* AS 3.4578 to produce compounds (**71–77)** and evaluated the biological effect of selected derivatives on increasing the lifespan of *Caenorhabditis elegans* [85].

**Fig. 16:**
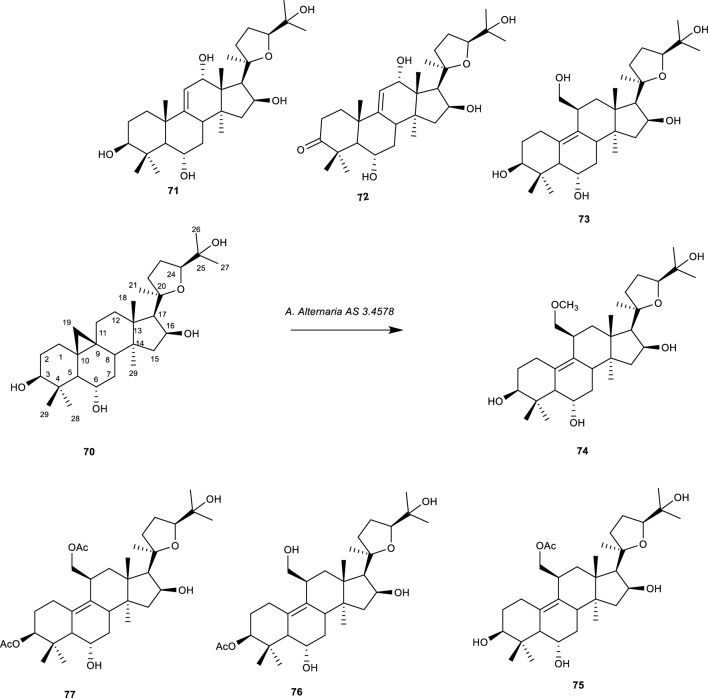
Biotransformation of cycloastragenol with *A. Alternaria* AS 3.4578. [86, 87]

These compounds were identified as (20R,24S)-3b,6a,12a,16b,25-pentahydroxy-20,24-epoxy-lanost-9(11)-ene (**71**), (20R,24S)-6a,12a,16b,25-tetrahydroxy-20,24-epoxy-lanost-9(11)-en-3-one (**72**), (20R,24S)-3b,6a,16b,19,25-pentahydroxy-ranunculan-9(10)-ene (**73**), (20R,24S)-3b,6a,16b,25-tetrahydroxy-19-methoxy-ranunculan-9(10)-ene (**74**), (20R,24S)-3b,6a,16b,25-tetrahydroxy-19-acetoxy-ranunculan-9(10)-ene (**75**), (20R,24S)-6a,16b,19,25-tetrahydroxy-3b-acetoxy-ranunculan-9(10)-ene (**76**), (20R,24S)-6a,16b,25-trihydroxy-3b,19-diacetoxy-ranunculan-9(10)-ene **(77)** as shown in Fig. 16.

Compounds **(70)** and **(71)** were transformed to possess the astragenol skeleton by the disappearance of the methylene group at C-19 and the formation of an olefinic bond at position C-9 and or C-11. Reduction of the OH group to carbonyl also occurred in compound **(72)**. Compounds **(73–77)** were obtained by rearrangement reaction on **(70)**. The formation of double bonds at C-9 and C-10 was observed in compounds **(73–77)**. While **(74**, **75**, and **76)** contain and acetoxy moiety at positions 19-OH, 3-OH, and 3-OH and 19-OH respectively. Compounds **(75** and **70)** were evaluated for their effects on the lifespan of *C*. *elegans* at a concentration of 50 mM and the result showed that **(70)** was more potent compared to **(75)** [86, 87]

**Fig. 17:**
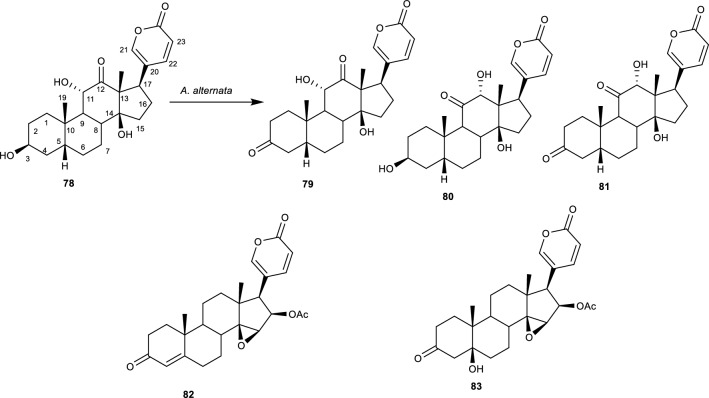
Biotransformation of arenobufagin with *A. alternata*. [53]

Arenobufagin **(78**) and cinobufotalin **(79**) are bufanolide steroids isolated from toad venom [88]. The dried skin secretions of giant toads (*Bufo gargarizans* *Cantor* or *Bufo melanostictus* Suhneider) are the source of toad venom (also known as Chan’su). For centuries, toad venom has been extensively utilized in TCM either on its own or in combination with other herbal components [53]. It has also been extensively used for treating pains, heart failure, sores, and different types of cancer [53]. Bufadienolides, which are the major bioactive compounds of Chan’su have gained a lot of attention lately because of their strong cytotoxic properties. However, due to their poor water solubility and strong cardiotoxicity, the majority of natural bufadienolides have limited clinical applications. As a result of this, much research is focused on structure modification using chemical or biological techniques to produce derivatives with enhanced cytotoxic activity or properties [53]. Hence, **(78)** and **(79)** were incubated with *A. alternata* and produced 11α, 14β-dihydroxy-3,12-dioxobufa-20,22-dienolide **(80)**, y-bufarenogin **(81)**, and 12α, 14β-dihydroxy-3,11-dioxobufa-20,22-dienolide (**82**), while metabolite **(79)** produced a 3-*oxo*-D^4^-derivative of cinobufotalin **(83)**, and 3-*oxo*-cinobufotalin **(84)**. Metabolites **(80)**, **(81)**, and **(82)** were formed by the oxidative dehydrogenation of **(77)** at positions C-3, C-11, and C-3 and C-11 respectively as shown in Fig. 17.

Metabolite **(83)** was biotransformed by the oxidative dehydrogenation of **(79)** at position C-3 and the elimination of OH at position C-5 to give a carbon–carbon double bond at C-4/C-5, while metabolite **(84)** was formed by the oxidative dehydrogenation of (**79)** at position C-3. The cytotoxic activity of compounds **(78)**, **(79)**, **(80)**, **(83)**, and **(84)** was evaluated against HepG2 and MCF-7 cell lines using MTT assay with etoposide as the positive control. The results indicated that the biotransformed metabolites had significant inhibitory activity against HepG2 and MCF-7 cell lines but were least active compared to **(78)** and **(79)** [53].

### Microbial ring cleavage reactions

**Fig. 18:**
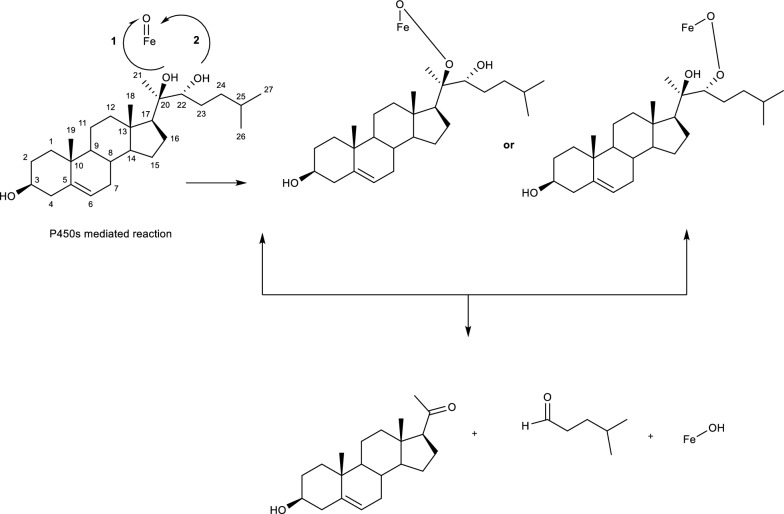
P450s mediated bond cleavage reaction mechanism [45]

In molecules bond cleavage is mostly initiated by a monooxygenase attack (introduction of two hydroxyl groups) to hydroxylase a substrate forming catechol intermediates, and subsequently followed by reacting with activated O_2_ to finalise the ring cleavage [45]. Other mechanisms that lead to bond cleavage include the use of CoA thioesters, ring activation and hydrolysis. One of the first studies in understanding bond cleavage in cyclic compounds includes a study by Sih et. al., (1966) where they explain the degradation of a steroid molecule leading to ring opening. [45] The authors also confirm the hydroxylation of the molecule followed by oxidation and some nonenzymatic rearrangement which they attribute to reverse aldolization. Other mechanisms involved in bond cleavage include the use of P450s, due to their ability to catalyze the formation and cleavage reactions of C–C bonds using the catalytic cycle modes (Fig. 1) [45]. Investigated the physiological C–C cleavage reactions that take place in the presence of P540s in the degradation of steroids as shown in Fig. 18 below. However, the mechanisms involved in these reactions are not yet understood [45].

**Fig. 19:**
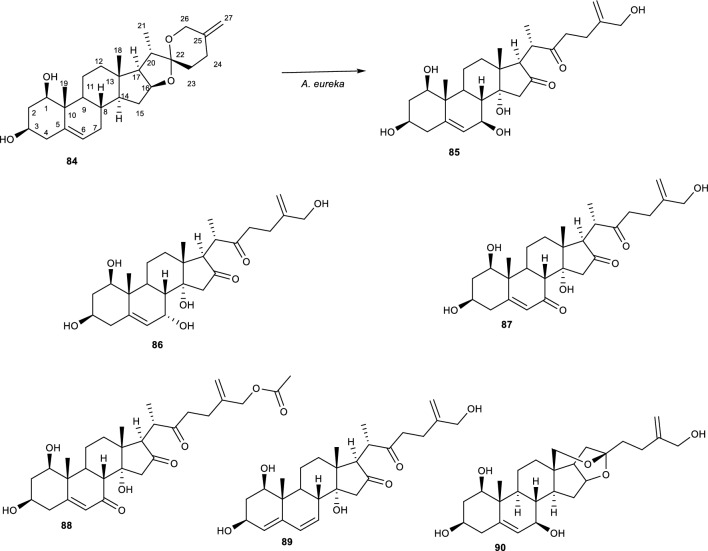
Biotransformation of neoruscogenin with *A. eureka* [44]

Neoruscogenin **(84)**, one of the spirostanol steroid isolated from the rhizomes of *Ruscus aculeatus* (Asparagaceae) is a bioactive ingredient of many herbal mixtures [44]. Its extract is used in alleviating arthritis related diseases such as chronic venous insufficiency, varicose veins, hemorrhoids, and orthostatic hypotension. To understand the metabolic pathway of **(84)** in mammals, Özçlnar *et. Al.,* (2018) investigated the biotransformation of **(84)** with endophytic fungus *A. eureka*. [89] The authors noted the ring cleavage capabilities of *A. eureka* involves specific mechanisms in support of the regioselectivity and stereoselectivity properties of P450s to convert a spiroketal system to its reported derivatives as shown in Fig. 19 [44].

**Fig. 20:**
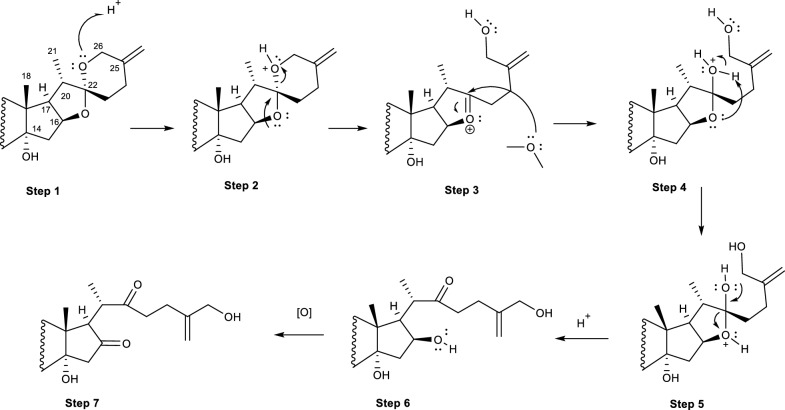
Proposed biosynthetic mechanism of spiroketal ring cleavage resulting to the cholestane-type framework [44]

Interestingly, the biocatalytic capabilities of *A. eureka* on **(84)** resulted in more than one reaction as the A and B ring system of the compound additionally experienced oxidation, hydroxylation, and dehydrogenation reactions. The resulting regiospecific reactions are observed at the C-5/6 olefinic bond due to an epoxidation reaction to obtain **(90)**. Additionally, the spiroketal hydrolysis reaction of compound **(84)** resulted in bond cleavage of the molecule to produce an acyclic side chain of compounds **(85 − 90)**. Methyl migration followed by acylation is also observed on the acyclic/cholestane- type steroidal framework of the molecule. Subsequently, a C-22(O)C-26 bond cleavage of the oxygen bridge produced a furostanol-type skeleton with its mechanism sown in Fig. 20. Additionally, the compound underwent some modification to yield a C-18(O)C-22 epoxide ring. [44].

### Acetylation reactions

**Fig. 21:**
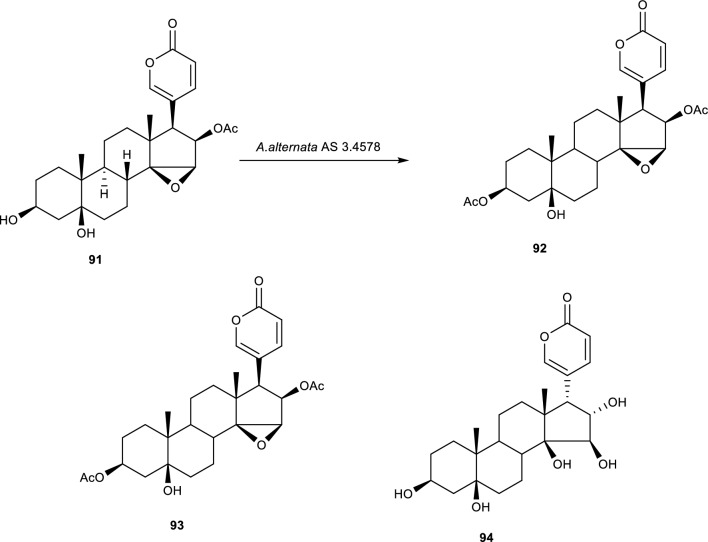
Biotransformation of cinobufotalin in the presence of *A. alternata* AS 3.4578 [88]

The biotransformation of cinobufotalin **(91)** in the presence of *A. alternata* resulted in three biotransformation products having underwent either acetylation or deacetylation at either position –3 or C-15 [88]. The biotransformation product 3-acetoxy-cinobufotalin **(92)** is shown to be a product of acetylation at C-3, notably the stereochemistry of this product was not transformed, similarly to the product 5β-hydroxy-deacetyl-cinobufagin **(93)** formed by converting the acetyl group at C-16 had no alterations in its stereochemistry suggesting that the reaction was only regioselective. However, the product 5β,15β,16α-trihydroxyl-17βH-bufalin **(94)** formed after deacetylation at position C-16 and the breaking of the epoxide at position C-14/15 to form the diol at positions C15/16 had a different stereochemistry at positions C-16 and C-17, suggesting that the fungi facilitated a stereospecific and regiospecific reaction to form the product Fig. 21 [88].

The ability of microorganisms to biotransform natural product compounds using specific enzymes allows compounds the opportunity to undergo regio and stereo-specific epoxidation. Microbial organisms such as those with P540s enzymes break the sp^3^ carbon bonds in natural occurring compounds to produce chiral epoxides. Such reaction allowed compounds such as limonene, terpinolene, phellandrene to form short-lived epoxide derivatives, however due to the high reactive nature of these intermediates, spontaneous conversions to diols via ring opening takes place suggesting epoxides produced during biotransformation are intermediates or needed for ring cleavage to produce diols.

### Ring expansion

**Fig. 22:**
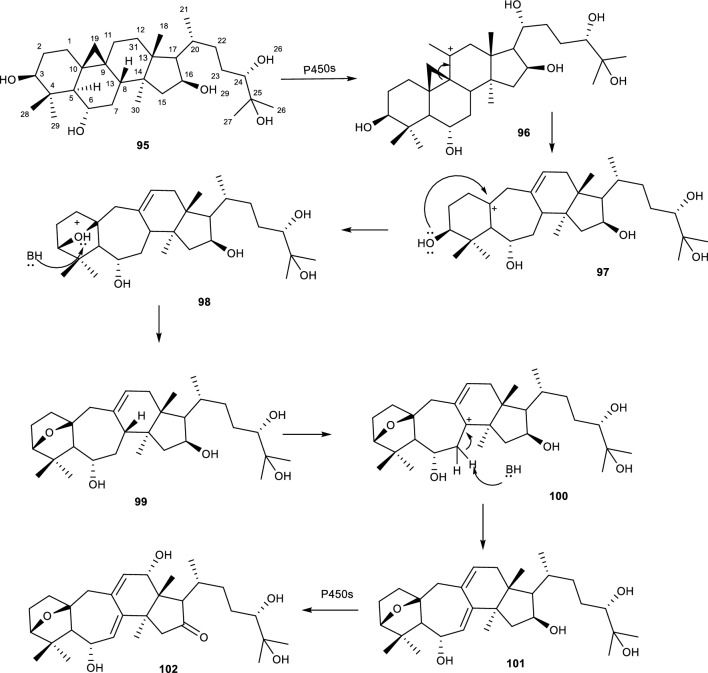
P450s mediated ring expansion mechanism [68, 69]

P450-mediated ring expansion reactions in natural product biosynthesis are important as they offer novel drug leads with interesting pharmacophores. These reactions are often at times initiated by abstraction of a hydrogen atom by compound 1 on a substrate to produce a radical molecule that is subsequently allowed to undergo a second hydrogen abstraction resulting in the desired ring expanded molecule. One of the few reported ring expansion reactions mediated by an *Alternaria* species makes use of *A. alternata. A. alternata* AS 3.4578 is reported to catalyze an unexpected ring expansion reaction that produces a rare 14(17) a-homo-18-nor-bufadienolide skeleton [68, 69]. The biocatalytic reaction is reported to be one of the challenging chemical approaches used to produce these metabolites shown in Fig. 22.

**Fig. 23:**
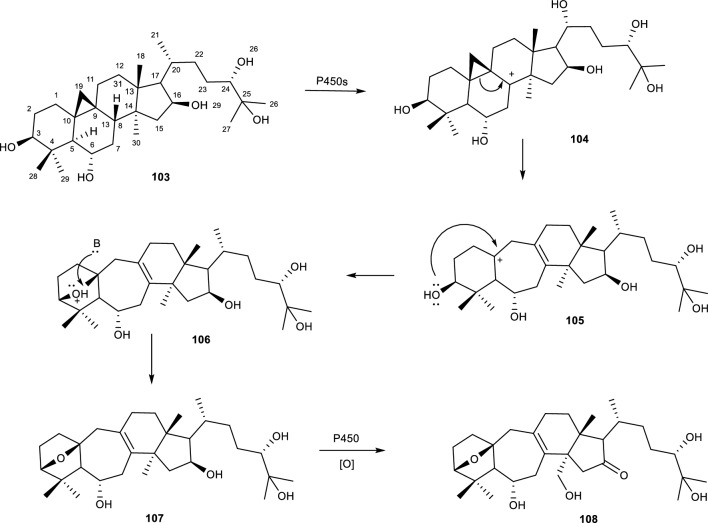
Biotransformation of cycloastragenol and *A. eureka* 1E1BL1 catalysed ring expansion mechanism. [85]

Another ring expansion mediated reaction by an *Alternaria* species is reported during the biotransformation of cycloastragenol with *A. eureka* 1E1BL. The fungal strain catalyzed ring expansion and epoxidation reactions on CCG **(106, 107 and 108)** to form 3(10)b-epoxy-9,10-seco-cycloartane structure as shown in Fig. 23. This is not the first time the B ring expansion reactions on cycloastragenol to give 9,10-secocycloartane skeleton. The Feng group also reported the production of such metabolites using the fungus *Syncephalastrum racemosum* AS 3.264 [85]. The authors noted that since cyclopropane rings behave similarly to a double bond in nature, The proposed that the C-1, C-5, C-8 and C-11 positions were subjected to allylic rearrangements. Additionally, the hydrogen abstraction from C-11 or C-8 by P450s forms a carbocation intermediate, which allows the migration of C-9/C-10 bond of the cyclopropane ring to afford 9,10-seco-cycloartane, C-8(C-9) or C-9(C-11) double bond and a stable carbocation at position C-10. The authors deduced that the transformation on ring A favours boat conformation, which rearranges C-3(OH) and C-10 with an observed adjacent localization that facilitates the nucleophilic attack of C-3(OH) to the carbocation (C-10) to give the 3(10)β-epoxy skeleton [85].

## Application of P450s catalysed biotranformation products

### Pharmaceutical application

The pressure on the pharmaceutical industry to find new molecules for various emerging diseases is forcing it to find new synthesis processes. New techniques have emerged, including nanotechnology, chemometrics, miniaturization, microdosing and high-throughput analysis. And with today's environmental demands, it has become essential to use synthesis processes with minimum environmental impact and maximum efficiency, hence the emergence of green chemistry. Green chemistry enables the production of fewer pollutants while ensuring high efficiency, and this is one of the characteristics of biotransformation. [91, 92]

In fact, biotransformation offers many advantages for the pharmaceutical industry. It can modify the chemical structure of many compounds, providing a wide range of new derivatives [93]. The molecules formed could have a higher activity than that of the parent molecule, a reversed activity or even a different and totally new activity. Furthermore, biotransformation could be responsible to produce compounds with lower toxicity than the parent molecule [94]. It is therefore an effective method for the discovery of new drug candidates [91]. In fact, the biotransformation of diosgenin (a steroidal sapogenin with multiple biological properties) was realized using *Rhodococcus erythropolis*. Four compounds were identified, two of which display potent cytotoxic activity against MCF-7, A549 and HepG2 cell lines. The remaining two compounds are novel molecules, one of which may have therapeutic potential in the treatment of hypertension, atherosclerosis, and chronic kidney disease. Moreover, by enhancing the expression of P450s enzymes, this compound was produced 2.9 times more [90].

Endophytic fungi and their P450s are commonly used for biotransformation reactions [89]. They offer a way of circumventing the problems associated with chemical synthesis [93]. They could also replace a series of reactions to synthesize the same compound [94]. Indeed, the production of the active form of vitamin D3 (1α,25-dihydroxyvitamin D3) from cholesterol requires some twenty steps, resulting in a minimal yield. Whereas the use of cytochrome P450 Vdh (CYP107BR1) from *Pseudonocardia autotrophica* NBRC 12743 enables its production in a single step (Table 3) [95].

**Table 3:** Endophytic fungi/P450s used to produce pharmaceuticals and chemical intermediates

Substrate	Substrate properties	Problem	Endophytic fungi/P450	Function	Produced compound	Compound properties	References
α-humulene	–	–	Engineered *Saccharomyces cerevisiae* CYP7BA1 from *Zingiber zerumbet*	Hydroxylation	8-hydroxy-α-humulene	Precursor of zerumbone (anti-inflammatory, anti-carcinogenic, and anti-obesity compound) Higher yield	[99]
Amorphadiene	–	Biosynthesis pathway with low yield of artemisinic acid	Engineered *Saccharomyces cerevisiae* CYP71AV1 from *Artemisia annua*	Three-step oxidation	Artemisic acid	Precursor of artemisinin (antimalarial drug) Higher yield	[100]
Artemisinin	Effective treatment for chloroquine resistant malaria	Low water solubility Creation of neurological lesions in the brainstem in animal models	*Eurotium amstelodami* A-51 *Aspergillus niger* VKM F-1119	Hydroxylation	5β-hydroxyartemisinin	Excellent water solubility and antimalarial profiles	[101]
Bufalin	Powerful bufadienolide against cancer cells	- Oxyfunctional sites limited to certain positions and difficult to obtain by chemical means - Difficulty in assessing the cytotoxic properties of oxygenated bufadienolides at other sites	*Mucor spinosus*	Hydroxylation	1β-hydroxyl bufalin 12β-hydroxyl bufalin	More polar bufadienolides Enhanced cytotoxic activity against BGC-823 and HeLa cell lines	[102]
Compactin	HMG-CoA reductase inhibitor	–	CYP105A3 or P-450_sca-2_ from *Streptomyces carbophilus*	Hydroxylation	Pravastatin	Potent and specific HMG-CoA reductase inhibitor (hypercholesterolemia therapy)	[103]
Cyclosporin A	Immunosuppressant drug	Provokes strong hair growth as a side effect	CYP-sb21 (CYP107Z14) from *Sebekia benihana*	Hydroxylation at the 4th *N*-methyl leucine	γ-hydroxy-*N*-methyl-l-Leu4-Cyclosporin A	Significantly reduced immunosuppressive activity Hair growth stimulation effect (great potential to treat alopecia)	[104]
Dihydromonacolin L	Lovastatin backbone	Unclear catalyzing system	*Aspergillus terreus* LovA (a cytochrome P450 gene)	4a,5-Dehydrogenation, 8-hydroxylation	Monacolin J	Pathway intermediate in the biosynthesis of lovastatin (hypercholesterolemic agent and a precursor to simvastatin, a cholesterol-lowering drug)	[105]
Noscapine	Anticancer drug	–	P450_BM3_ (CYP102A1) in *Bacillus megaterium*	*N*-demethylation	*N*-nornoscapine	Precursor for *N*-modified noscapine analogues with stronger antitumour activity	[106]
Papaverine	Prevents calcium release from internal stores, increases cGMP and cAMP levels in smooth muscle, and promising anti-tumor agent	Difficulty of selective *O*-demethylation at an industrial scale	*Escherichia coli* BL21(DE3) CYP105D1 from *Streptomyces griseus*	Demethylation	6-*O*-demethyl-papaverine	Less cytotoxic than papaverine	[107]
Progesterone	–	–	*S. cerevisiae* expressing CYP68J5_fg from *Fusarium graminearum*	12β- and 15α-hydroxylase	12β,15α-dihydroxyprogesterone 12β-hydroxyprogesterone 15α-hydroxyprogesterone	Identification of a new fungal P450 enzyme with 12β-hydroxylase activity (potential application for the synthesis of C12-hydroxylated steroid drugs)	[108]
Rabeprazole sulfide	One of the active metabolites of rabeprazole	High cost of rabeprazole (drug for peptic disorders treatment)	CYP102A1 WT cloned and expressed in *Escherichia coli*	*O*-desmethylation	Desmethyl rabeprazole sulfide	Product of the metabolism of rabeprazole sulfide in humans	[109]
Resibufogenin	Significant cytotoxicity against several cell lines	Inability to study the activity of some analogues, and explore their structure–activity relationship	*Nocardia* sp. NRRL 5646	14β,15β-epoxy ring cleavage and a regio-selective acetoxylation	3-acetyl 15β-hydroxyl bufotalin	Increased cytotoxicity against HepG2, BGC823 and HeLa cell lines	[110]
Taxadiene	–	–	*Escherichia coli* with optimizing P450 expression	5α-Hydroxylation	Taxol	Higher yield of taxol	[111]
Vitamin D_3_	Prohormone that plays a crucial role in immunity, bone metabolism, and control of cell differentiation and cell proliferation in mammals	Biologically inactive The physiologically active form (1a,25-dihydroxyvitamin D3) can be chemically synthesized from cholesterol via a complex and quite expensive procedure (about 20 steps and very low yield)	P450 Vdh (CYP107BR1) from *Pseudonocardia autotrophica* NBRC 12743 introduced into *Escherichia coli* BL21(DE3)	25-Hydroxylation 1α- Hydroxylation	25-hydroxyvitamin D3 1α, 25-dihydroxyvitamin D3	Treatment of chronic renal failure, osteoporosis, hyperparathyroidism, and psoriasis	[111]

P450s can also improve the pharmacokinetic parameters of several compounds, including absorption and solubility. They catalyze the production of compounds from exogenous molecules, whose structure has undergone minor modification to increase their polarity (see examples in Table 3). Biotransformation can therefore play an important role in increasing the bioavailability of certain compounds [91].

The wide range of enzymes in endophytic fungi catalyze chemical reactions necessary for their growth and reproduction that are often like human mono-oxygenase systems [93]. For the pharmaceutical industry, drug design requires the identification of potential intermediate compounds in mammalian metabolism [93, 94]. A good example is that of phloretin (a polyphenolic compound abundant in apples) that has several benefits for human health. When studying its metabolic pathway in humans, the roles of hepatic P450s were examined. Among the main metabolites whose reactions were catalyzed by CYP3A4 and CYP2C19 was 3-hydroxyphloritin. Phloretin was also found to be a potent inhibitor of testosterone 6β-hydroxylation activity catalyzed by CYP3A4. As CYP3A4 is responsible for the metabolism of many drugs in the liver and small intestine, phloretin consumption could affect this metabolism, and even cause significant side effects [96].

P450s are part of a research field that continually provides new enzymes and mutants that produce metabolites with potential use as new drugs, or as precursors for the synthesis of other compounds [97]. New enzyme variants are continually being created through metabolic and protein engineering. Moreover, the introduction of entire new biosynthetic pathways into host organisms has an impact on the quantity of metabolites produced with new or enhanced activities (Table 3) [98].

### Agricultural application

Biotransformed compounds applications in agriculture has emerged as a promising strategy promoting sustainable and eco-friendly practices [111]. Biotransformation, driven by diverse microorganisms and enzymes, allows in one hand the production of new compounds with potential uses in improving plant growth, pest control and overall plant bioprotection. On the other hand, biotransformation became a significant element in bioremediation of polluted agricultural soils [112]. Several studies have shown that microorganisms become a significant contributor in the biotransformation and detoxification of harmful compounds such as pesticides as noted in Table 4. These chemical compounds can persist in the agricultural soils causing serious problems. Xia group demonstrated that the residual presence of the herbicide 2,4-dichlorophenoxyacetic acid in agricultural soils exhibits considerable phytotoxic effects on maize crops [114]. In this case, biotransformation using the bacterial strain *Achromobacter* sp. LZ35 has proven to be an effective strategy for alleviating the phytotoxicity associated the herbicide 2,4-dichlorophenoxyacetic acid and its residuals [114].

**Table 4:** Microbial biotransformation of pesticides and their associated byproducts

Parental substances	Microbial strain	Source	Byproducts	Biotransformation process	References
1,1,1-trichloro-2,2-bis(p-chlorophenyl) ethane (4,40-DDT)	*Corynebacterium sp.*	Sandy loam soil	DDD (1,1-dichloro-2,2-bis(p-chlorophenyl)ethane) and DDE (1,1-dichloro-2,2-bis(p-chlorophenyl)ethylene)	Biodegradation	[120]
2,4-dichlorophenoxyacetic acid (2,4-D)	*Achromobacter sp. LZ35*	Contaminated soil	Not specified	Biodegradation	[113]
2-methyl-4-chlorophenoxy acetic acid (MCPA)	*Achromobacter sp. LZ35*	Contaminated soil	Not specified	Biodegradation	[113]
6:2 fluorotelomer sulfonate	*Desulfobacterota*	Contaminated soil	C4–C7 perfluoroalkyl carboxylates	Desulfonation	[121]
Bifenthrin	*Bacillus pseudomycoides 3RF2C*	Leaves of orange trees	Not specified	Biodegradation	[122]
Butralin	*Sphingopyxis sp. strain HMH*	Agricultural soil	5-(tert-Butyl)-3 -nitrobenzene-1,2-diamine and butanone	Nitroreduction and N-dealkylation	[123]
Chlorpyrifos	*Hortaea sp. B15*	Not specified	3,5,6-trichloropyridin-2-ol and 2-pyridinol	Biodegradation	[124]
Dieldrin	*Penicillium miczynskii*	Marine sponge	Not specified	Biodegradation	[125]
Endosulfan	*Bacillus subtilis Aspergillus niger*	Not specified	Endosulfan diol, ether, hydroxyether, and lactone	Hydrolysis and oxidation	[126]
Esfenvalerate	*Penicillium raistrickii, Aspergillus sydowii*	Marine sponge	2-(4chlorophenyl)-3-methylbutyric acid, 3-phenoxybenzyl alcohol, 3-phenoxybenzaldehyde, 3-phenoxybenzoic acid	Biodegradation	[127]
Fipronil	*Bacillus amyloliquefaciens RFD1C*	Leaves of orange trees	Not specified	Biodegradation	[122]
Hexabromocyclododecanes	*Sphingobium chinhatense IP26*	Contaminated soil	Pentabromocyclododecanol isomers, 11 tetrabromo- cyclododecadiols and 3 tribromocyclododecatriols	Dehalogenation	[128]
Lindane	*Paracoccus sp. NITDBR1*	Agricultural soil	Not specified	Biodegradation	[129]
Meyhyl parathion	*Fusarium proliferatum*	Ascidian Didemnum ligulum	Not specified	N-acetylation and bioconjugation	[130]
Thiencarbazone-methyl	*Streptococcus pneumoniae, Escherichia coli, Streptococcus pyogenes*		Methyl-4-iso cyanatosulfonyl-5-methylthiophene-3-carboxylate, 4-methanesulfonyl-benzenesulfonic acid amide and methyl 3-sulfamoylthiophene-2carboxylate	Biodegradation	[131]

Recently, Zhang Y. and coworkers revealed the ability of the bacterial strain *Brevibacillus parabrevis* to biotransform deltamethrin pesticide through a successive chemical reaction (e.g., oxidation, hydrolysis, and decarboxylation) until a complete mineralization into CO_2_ and H_2_O [114]. Besides the crucial role of biotransformation in neutralizing hazardous compounds, this process also extends to using microorganisms as a novel and ecofriendly approach to generate natural products with significant agrochemical properties that can contribute to plant bioprotection. It has been demonstrated that a marine fish-derived fungus*, Chaetomium globosum,* can biotransform 1-Methyl-L-tryptophan into new indole alkaloids including chaetoindolone A and Chaetogline A. These compounds have shown an inhibitory effect of the rice-pathogenic bacteria *Xanthomonas oryzae* and *Sclerotinia sclerotiorum*, a fungal pathogen causing rape sclerotinia rot, respectively [115]. Moreover, biotransformation process has been exploited to improve the efficacy of the biopesticides; α- and β-pinenes using the mycelium of *Pleurotus* [116]*.*

Further extending the scope of producing natural products inhibiting plant pathogenic microorganisms through biotransformation. This process offers an environmentally friendly alternatives to conventional pesticides. Wang et al. Demonstrated that certain *Pseudomonas* species in the rhizospheric soil, have showed the potential in the biotransformation of the allelochemical (−)-catechin that has been identified as the major allelochemical in the leaves of *Rhododendron formosanum.* The biotransformation products were identified as protocatechuic acid which has a higher allelopathic effect than catechin [117]. Enhancing the allelopathic activity of plants could potentially aid in plant bioprotection [118, 119] and achieve sustainable agricultural practises [113, 120].

Microbial biotransformation of chemical compounds has not only showed an improvement in plants' defensive mechanisms but also it becomes a sustainable strategy in the promotion of plant growth and boosting agricultural productivity. The same microbial reactions that neutralize hazardous compounds and produce biopesticides can be harnessed to enhance plant growth. Recently, Gamel and others explored the biotransformation of *Acacia nicolotica* metabolites by the fungal strain *Aspergillus sublivaceus* in promoting *Lupinus termis* yield. Natural products resulted from this biotransformation, showed a significant improvement in seed yield attributes and biochemical contents, including increases in total nitrogen, amino nitrogen, glucose, and protein levels of the seeds [111]. Another study conducted by Wang and coworkers revealed the potential application of biotransformed biotite in agriculture using the common fungus *Aspergillus niger.* This fungal strain showed a significant ability in weathering biotite, and release key nutrients like potassium, which are essential for plant growth [132, 133]. These findings confirm the promising role of biotransformation as an ecofriendly strategy within sustainable agricultural practices.

### Food sector application

Despite the considerable potential of biotransformation in agricultural and medical sectors, it remains underexplored in the food industry. However, the limited number of studies showed a promising pathway for biotransformation applications in the food sector. This process has been shown to remarkably improve the nutritional and health-promoting properties of food products. Ferreira et. al. [136] have explored the use of enzymatic biotransformation in enhancing the chemopreventive potential of orange juice [134]. The authors demonstrated how the enzymatic biotransformation of orange juice polyphenols, using tannase enzyme, could modify their biological activity. This modification has not only changed the polyphenolic composition of orange juice but also significantly enhances its bioactivity. The biotransformation process leads to an increase in the antioxidant capacity of the juice, which is crucial in preventing oxidative stress linked to chronic diseases like cancer.

Another study conducted by Mazlan group has confirmed the value of biotransformation in enhancing nutritional and medicinal properties of food [135]. This investigation showed the ability of the bacterial strain *Lactobacillus plantarum* BET003 in the biotransformation of *Momordica charantia* (bitter melon) juice and reducing its bitterness and sugar content while producing beneficial aglycones and metabolites. The fermented juice exhibited significant anti-diabetic properties by inhibiting α-glucosidase activity, suggesting a potential therapeutic application in managing diabetes [135]. A recent study demonstrated the significant potential of propionic acid bacteria in the biotransformation of soy (tofu) whey, enhancing the content of short-chain fatty acids and vitamin B12 in this product [136]. Moreover, the process resulted in the bioconversion of isoflavone glycosides to aglycones, indicating a potential application in creating functional beverages or ingredients and the possibility of biofortifying food products and contributing to sustainable food processing practices.

Besides the significant role of microbial biotransformation in nutritional and health-promoting properties of food products, it can present a valuable tool in food processing to create new flavors and improve the sensory qualities of food products. Recently, Fei and coworkers highlighted the role of biotransformation in enhancing food flavors and producing unique fermentation flavors and active ingredients [137]. Their study revealed how the fermentation of bamboo leaf juice using the probiotic bacterium *Streptococcus thermophilus* can significantly improve the sensory properties of the juice. This process successfully reduced undesirable flavors, such as bitterness and astringency, while increasing desirable fermentation flavors, contributing to the overall palatability and acceptability of the product.

## Conclusions and future directions

In this work, all the studies focusing on the microbial biotransformation of secondary metabolites by *Alternaria* fungi were extensively evaluated and reported. *Alternaria* species make use of the P450s to facilitate different reaction mechanisms during biotransformation studies. These fungal microorganisms especially, *A. alternata, A. eureka,* and *A. longipes* are mainly reported in most biotransformation studies and facilitate reactions such as hydroxylation, oxygenation, ring cleavage, acylation and more. Additionally, these reactions have been shown to be stereospecific, regioselective and chemoselective to produce novel biotransformation products with improved bioactivity. Hence, the biotransformation of natural product-based compounds using other *Alternaria* species can be explored as these microorganisms have been proven to be an important tool to reach specific reactions and functionalization of deactivated carbons. Finally, the knowledge of both the biological activities of biotransformed compounds and the role of these compounds in agriculture and food application may inspire the development of new products with improved selectivity resulting in food, biostimulants and medicinal mixture or new drug leads with enriched properties. Of note is the limited information on the use of *Alternaria* species are non-existent, hence the need for more studies and their application in agriculture and food.

## Data Availability

All the data and materials provided in the manuscript are obtained from included references.
